# Morphological Spectrum of Gastritis in Endoscopic Biopsies and Its Association With Helicobacter pylori Infection

**DOI:** 10.7759/cureus.43084

**Published:** 2023-08-07

**Authors:** Aribah Atiq, Muhammad Moseeb Ali Hashim, Faria W Khan, Azra Bashir, Asma Zafar, Anum Jamil, Akhtar S Chughtai

**Affiliations:** 1 Pathology, Chughtai Institute of Pathology, Lahore, PAK; 2 Histopathology, Chughtai Institute of Pathology, Lahore, PAK

**Keywords:** sydney classification, antrum, gastritis, helicobacter pylori, chronic gastritis

## Abstract

Introduction

*Helicobacter pylori* (*H. pylori*) infection is the most common cause of gastritis. The consequences of the persistent infection range from acute ulceration to the development of gastric neoplasia. The purpose of the study is to determine the histopathological pattern of gastritis in gastric biopsies and its association with *H. pylori*.

Materials and methods

This is a retrospective study of all the gastric biopsies received in the Department of Histopathology, Chughtai Institute of Pathology, Lahore, Pakistan from January 12, 2021 to April 10, 2021. Sections were cut from formalin-fixed, paraffin-embedded (FFPE) tissue blocks. Slides were stained with routine, special and immunohistochemical stains. The cases were then blindly reviewed by two pathologists with a special interest in Gastrointestinal Pathology. Microscopic features based on updated Sydney classification were recorded. The collected data were then analyzed by using SPSS version 20 (IBM Corp., Armonk, NY).

Results

A total of 429 gastric biopsies were examined. Out of which, 202 (47.1%) were male and 227 (52.9%) were female with a female-to-male ratio of 1.2:1. Their ages ranged from 12 to 100 years and a peak in the fourth decade of life with a mean age of 43 years (median age 49 years). Among gastritis, activity was seen in 194 (45%) and chronicity in 237 (55%) cases. *H. pylori* was seen in 174 (40.5%) cases and there was a strong statistically significant association between *H. pylori* infection and severity of gastritis (p<0.001). Other parameters of Sydney classification, such as atrophic changes, were seen in 144 (33%) cases, and intestinal metaplasia in 10 (2.3%) cases.

Conclusion

*H. pylori* organism was the most common cause of gastritis in our environment. The severity of gastritis is directly related to the *H. pylori* infection. If neutrophils are identified on surface epithelium, then *H. pylori* should be searched with increased attention on morphology and/or on immunohistochemical stain of *H. pylori*. Chronic inflammation and *H. pylori* density can also guide treatment which is necessary to avoid complications.

## Introduction

Gastritis is usually described as the inflammation of gastric mucosa. It can have various etiologies, but the most common cause is *Helicobacter pylori *(*H. pylori*). *H. pylori *is a flagellated, curved, gram-negative rod that is oxidase-positive, catalase-positive, and urease positive. It colonizes mainly the antrum of the stomach but is also seen in the body; causes gastritis and peptic ulcers. It has adaptive features for the colonization of the human stomach and is present in about half of the human population [1]. The prevalence rate of *H. pylori* and its associated diseases has been highly inconsistent worldwide. It causes a wide spectrum of morphological gastric pathologies such as mild active gastritis, moderate active gastritis, severe active gastritis, atrophy, and intestinal metaplasia [2,3]. The infection has been prevalent in 17% of gastric ulcer cases and more than 75% of duodenal ulcer cases [4]. It is the major cause of adenocarcinoma of the distal stomach and B-cell mucosa-associated lymphoid tissue (MALT) lymphoma. It is also linked with extra gastric manifestations including iron deficiency anemia, idiopathic thrombocytopenic purpura, and vitamin B12 deficiency (commonly but not exclusively seen in the setting of autoimmune atrophic gastritis). The infection is asymptomatic in the vast majority of cases; more serious outcomes occur in only 10%-15% of the cases [5]. Therefore, all cases of *H. pylori* must be treated to prevent these complications. The biopsy is the gold-standard investigation to diagnose gastritis, neutrophilic infiltration, the density of *H. pylori* colonization, acute or chronic inflammation, intestinal metaplasia, and malignancy. With these considerations in mind, this study attempts to describe the morphological alterations according to the updated Sydney classification, the prevalence of *H. pylori,* and its association with the density of inflammation.

## Materials and methods

This is a retrospective cross-sectional study conducted at the Histopathology Department, Chughtai Institute of Pathology, Lahore, Pakistan from January 12, 2021 to April 10, 2021. This study was approved by the institutional board review of the Chughtai Institute of Pathology (IRB no CIP/IRB/1145). The study was conducted following the principles of the Declaration of Helsinki. The gastric biopsies of the patients undergoing upper gastrointestinal endoscopic evaluation were included in this study. Biopsies in which the site is ambiguous and biopsies with poor preservation were excluded from the study. The non-probability consecutive sampling technique was used. Biopsies of male and female patients of all ages were included in this study. Biopsy specimens were fixed in 10% formalin. All of them were routinely processed and paraffin blocks were prepared. 3-4 μm thick sections were cut from the blocks. Two slide sets (with three levels on each slide) were made from each specimen, and both sections were routinely stained with hematoxylin and eosin (H&E). All slides were evaluated under a light microscope by two Gastrointestinal Pathologists. The histomorphology of sections was primarily studied on routine H&E-stained sections. *H. pylori* immunohistochemical (IHC) stain was studied for the identification of *H. pylori* in each case. Dako polyclonal, ready-to-use, *H. pylori* antibody was used for this purpose. The antigen retrieval time was 20 minutes and Dako Autostainer link 48 was used for staining purposes. A quantitative assessment method was used for the evaluation of *H. pylori* on IHC. Mild colonization was defined as scattered organisms found in less than one-third of the surface epithelium. When there were large clusters or continuous layers of organisms seen over two-thirds of the surface, that was defined as severe colonization. Moderate colonization was the intermediate state between the above two. The demographical variables of the patients from which the sample is drawn were noted, which include age, gender, clinical history, and histological diagnosis. The data were entered and analyzed using Statistical Package for Social Sciences (SPSS) version 29 (IBM Corp., Armonk, NY).

## Results

A total of 429 gastric biopsies were studied which met the inclusion criteria. 202 (47.1%) samples were obtained from males and 227 (52.9%) samples were obtained from females with female to male ratio of 1.12:1. The histopathological parameters were studied according to the updated Sydney Classification as shown in Table 1.

**Table 1:** Histopathologic characteristics according to updated Sydney classification

Histopathological features	Grade			Total (%)
	Mild (n %)	Moderate (n %)	Severe (n %)	
H. pylori	144 (79.2%)	22 (15.3%)	8 (5.6%)	174 (40.5)
Activity	131 (60.6%)	63 (29.2%)	22 (10.2%)	194 (45)
Chronicity	225 (94.9%)	11 (4.6%)	1 (0.4%)	235 (55)
Atrophic Changes				144 (33)
Intestinal Metaplasia				10 (2.3)

Among 429 cases, 144 (33%) cases showed atrophic changes, as shown in the table above. However, 174 (40.5%) cases showed *H. pylori*. Activity was seen in 194 (45%) of cases and chronicity in 235 (55%) cases. Only 10 (2.3%) cases showed intestinal metaplasia.

Out of 174 *H. pylori*-positive biopsies as shown in Table 2, mild colonization was seen in 144 (79.2%) samples, moderate colonization was seen in 22 (15.3%), and severe colonization was seen in eight (5.6%). In 174 *H. pylori*-positive gastric biopsies, mild active gastritis was seen in 75 (43.1%) biopsies, moderate active gastritis was seen in 34 (19.5%) biopsies and severe active gastritis was seen in 11 (6.3%) biopsies.

**Table 2:** Distribution of histomorphological features among H. pylori positive and negative groups

H. pylori status (n)	Chronic Inflammation (Mononuclear infiltration) (n %)			Neutrophilic Activity (n %)		
	Mild	Moderate	Severe	Mild	Moderate	Severe
Positive (174)	139 (79.8%)	8 (4.5%)	0 (0%)	75 (43.1%)	34 (19.5%)	11 (6.3%)
Negative (255)	75 (29.4%)	3 (1.1%)	1 (0.3%)	50 (19.6%)	27 (10.5%)	10 (3.9%)

In *H. pylori*-negative gastric biopsies, mild active gastritis was seen in 50 (19.6%), moderate active gastritis was seen in 27 (10.5%), and severe active gastritis was seen in 10 (3.9%). Active gastritis with neutrophilic activity was seen in 194 (45%) gastric biopsies and chronic gastritis was seen in 235 (55%) gastric biopsies. There was a strong statistically significant association between *H. pylori* infection and the severity of gastritis (p<0.001).

## Discussion

The most common histopathological diagnosis in gastric biopsies is chronic gastritis. *H. pylori *is considered the most common etiological agent. Prevalence generally varies with the method of investigation employed, according to the widely accepted concept. The most commonly employed invasive method of *H. pylori* detection in the clinical setting is the histopathological examination. Other methods that have been used for the detection of *H. pylori* in the tissue sections of paraffin-embedded gastric mucosa specimens are H&E, Giemsa, silver staining, and immunohistochemistry. An adequate number of gastric biopsies, rightly chosen biopsy sites, or the skill of the pathologist is required for the accurate detection of *H. pylori*. Using histopathology as a method of investigation also gives you information about morphological changes in the gastric mucosa demonstrating gastritis, intestinal metaplasia, dyspepsia, atrophy, or malignancies.

**Figure 1:**
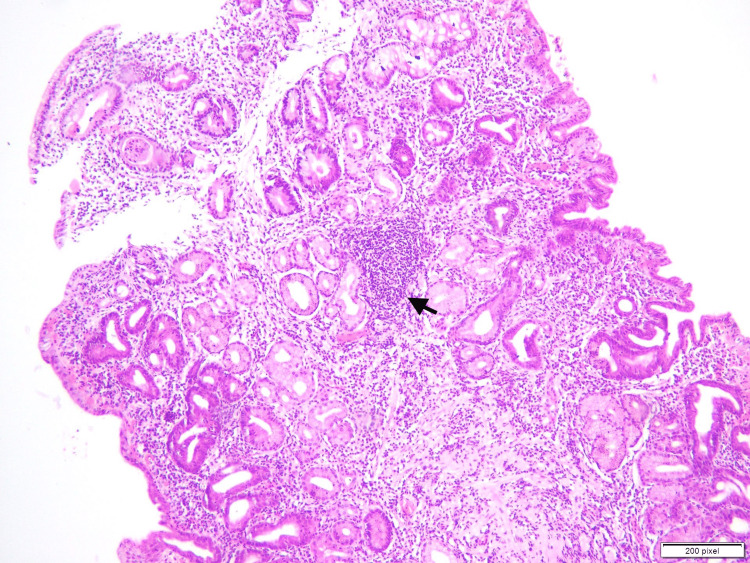
Low power view (10x) of gastric antral mucosa showing chronic inflammation and lymphoid aggregate (arrow) The figure is taken from one of the case included in the study (Author: Aribah Atiq) The software which is used for taking pictures is calibrated at “200 pixels”

**Figure 2:**
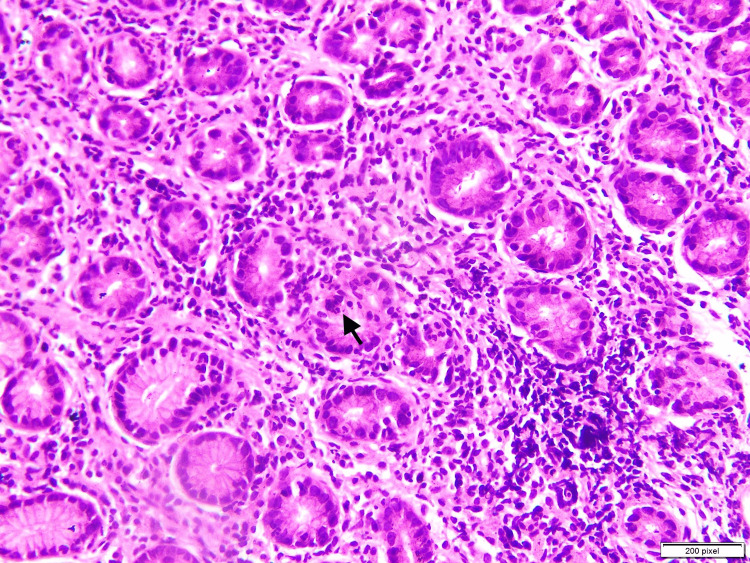
High power magnification (40x) of gastric antral biopsy showing neutrophilic infiltration of the gastric pits (arrow), defined as activity The figure is taken from one of the cases included in the study (Author: Aribah Atiq) The software which is used for taking picture is calibrated at “200 pixel”

Antrum is the most suitable site for the evaluation of gastric biopsies and detection of *H. pylori* (Figures 1, 2). Eriksson et al. in a study have also suggested that the most likely site of histopathological findings in gastritis is Antrum [6]. The most common site in our study was also antrum and this finding is in keeping with various other studies like Garg et al. [7] Park et al. [8], and Dhakhwa et al. [9].

Chronic gastritis is defined as the infiltration of mononuclear cells and plasma cells (Figure 1). Chronic gastritis is seen in 235 (55%) cases in the present study. The severity of gastritis is mild. These findings are synonymous with various other studies. One example is a study conducted by Garg et al. and he also found that the grade of inflammation in majority of the studies was mild [7]. And it was also suggested in the study that severity of gastritis is directly proportional to the degree of colonization of *H. pylori,* which was also in keeping with the results of our study.

**Figure 3:**
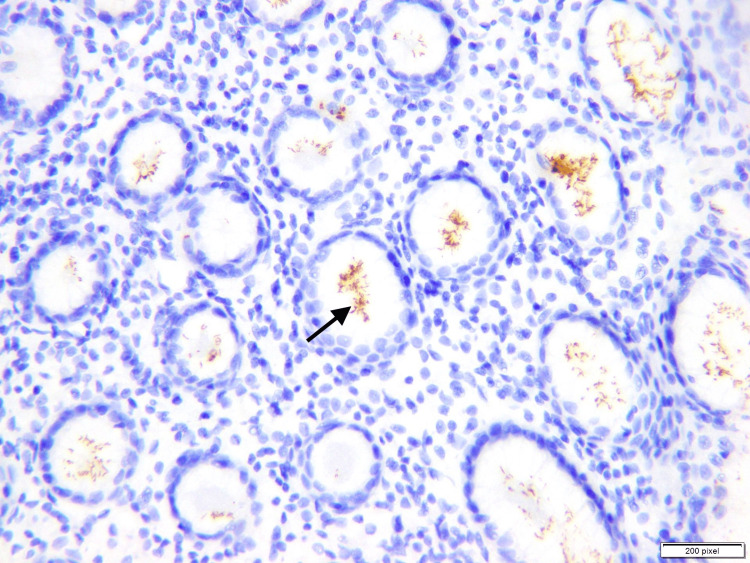
High power view (40x) showing moderate to severe (arrow) colonization of H. pylori on immunohistochemical stain The figure is taken from one of the cases included in the study (Author: Aribah Atiq) The software which is used for taking pictures is calibrated at “200 pixels”

In our study, 143 (33.3%) cases out of 429 (100%) cases showed *H. pylori* and these cases stained positive with *H. pylori* IHC stain, as shown in Figure 3. Here we have a slightly different result here, the prevalence of *H. pylori* calculated by Grag et al. is 43.66% and Hassan et al. is 93.7% [7,10]. The severity of gastritis is directly proportional to the degree of colonization of *H. pylori*. Milder *H. pylori* colonization, milder gastritis, and moderate gastritis is seen in patients with a moderate degree of colonization and severe gastritis is seen in patients with high-density colonization of *H. pylori*. Similar results were found in other studies [8,11-13]. However, the study results are contrary to the study by Park et al. [8], which there is no association between the degree of colonization of *H. pylori* and the severity of gastritis. But in our study, there is a strong statistically significant association between the degree of gastritis and *H. pylori*.

The variations in biopsy sampling sites are responsible for varying prevalence of *H. pylori* intragastric colonization. Various other factors that contribute to the varying prevalence include prior treatment with proton pump inhibitors or anti-*H. pylori* antimicrobial agent and immune-host response. Of note, *H. pylori* colonization is best assessed in the gastric antrum.

Activity, which is usually defined as neutrophilic infiltration of the pits (Figure 3), is seen in 194 biopsies (45%) out of a total of 429 (100%) biopsies. As compared to the other studies conducted by Park et al. (78.75%) and Hussein et al. (84%), our value is much lower. However, the result of this study exceeds the numbers of neutrophilic infiltration in studies by Garg et al. and Maharjan et al., in which the results are (33.33%) (31.84%), and (33.6%), respectively [7,10,14]. Hence, there is no statistically significant association between activity and the colonization of *H. pylori.* Ten (2.3%) cases showed intestinal metaplasia in our study. 

In discordant cases with high degree of gastritis and intestinal metaplasia, IHC stains can be used to search for *H. pylori* or other Helicobacter species such as *H. helmanii*. Immunohistochemistry requires careful consideration of the antibodies used, potential cross-reactivity, and interpretation. However, IHC staining is just one tool in the diagnostic process. It should be used in conjunction with endoscopy results, clinical findings, and other diagnostic tests to make an accurate diagnosis of *H. pylori* infection.

The limitation of this study is that biopsies with the ambiguity of the site are excluded, and only antral biopsies are included. Biopsies with poor preservation (3% of cases) are also excluded from the study. The assessment of gastric inflammatory conditions would have been improved if the updated Sydney system guidelines were followed [15].

## Conclusions

This study concludes that the most common type of gastritis prevalent is chronic gastritis and is associated with *H. pylori*. Moreover, the degree of colonization of *H. pylori* is associated with the severity of gastritis. If neutrophils are seen on surface epithelium, an extra effort should be made to search for *H. pylori. *When standard H&E staining may not provide a definitive diagnosis, immunohistochemistry is a valuable tool for the evaluation of *H. pylori*.

Chronic inflammation and *H. pylori* density can guide treatment and follow-up to avoid complications. The diagnostic sensitivity can be improved if the proper guidelines are followed to take biopsies from multiple sites.
